# High-Risk Human Papillomavirus E7 Proteins Target PTPN14 for Degradation

**DOI:** 10.1128/mBio.01530-16

**Published:** 2016-09-20

**Authors:** Elizabeth A. White, Karl Münger, Peter M. Howley

**Affiliations:** a Department of Microbiology and Immunobiology, Harvard Medical School, Boston, Massachusetts, USA; b Department of Developmental, Molecular and Chemical Biology, Tufts University School of Medicine, Boston, Massachusetts, USA

## Abstract

The major transformation activity of the high-risk human papillomaviruses (HPV) is associated with the E7 oncoprotein. The interaction of HPV E7 with retinoblastoma family proteins is important for several E7 activities; however, this interaction does not fully account for the high-risk E7-specific cellular immortalization and transformation activities. We have determined that the cellular non-receptor protein tyrosine phosphatase PTPN14 interacts with HPV E7 from many genus alpha and beta HPV types. We find that high-risk genus alpha HPV E7, but not low-risk genus alpha or beta HPV E7, is necessary and sufficient to reduce the steady-state level of PTPN14 in cells. High-risk E7 proteins target PTPN14 for proteasome-mediated degradation, which requires the ubiquitin ligase UBR4, and PTPN14 is degraded by the proteasome in HPV-positive cervical cancer cell lines. Residues in the C terminus of E7 interact with the C-terminal phosphatase domain of PTPN14, and interference with the E7-PTPN14 interaction restores PTPN14 levels in cells. Finally, PTPN14 degradation correlates with the retinoblastoma-independent transforming activity of high-risk HPV E7.

## IMPORTANCE

High-risk human papillomaviruses (HPV) are the cause of cervical cancer, some other anogenital cancers, and a growing fraction of oropharyngeal carcinomas. The high-risk HPV E6 and E7 oncoproteins enable these viruses to cause cancer, and the mechanistic basis of their carcinogenic activity has been the subject of intense study. The high-risk E7 oncoprotein is especially important in the immortalization and transformation of human cells, which makes it a central component of HPV-associated cancer development. E7 oncoproteins interact with retinoblastoma family proteins, but for several decades, it has been recognized that high-risk HPV E7 oncoproteins have additional cancer-associated activities. We have determined that high-risk E7 proteins target the proteolysis of the cellular protein tyrosine phosphatase PTPN14 and find that this activity is correlated with the retinoblastoma-independent transforming activity of E7.

## INTRODUCTION

Human papillomaviruses (HPV) are the etiologic agents for cervical cancer, many other anogenital cancers, and an increasing proportion of oropharyngeal cancers (1). The 8-kbp double-stranded DNA HPV genome encodes the factors required to reprogram the infected host cell and support differentiation-dependent virus replication in stratified squamous epithelial cells. Of the more than 200 different HPV types that have been identified, there are 13 to 15 “high-risk” genus alpha HPV types that have been associated with anogenital and oral cancers (1–3). Dysregulated expression of high-risk HPV E6 and E7 oncoproteins can result in cellular immortalization, transformation, and eventually cancer. The E6 and E7 proteins encoded by the non-cancer-associated HPVs also have essential roles in the virus life cycle but are generally not active in transformation assays.

The high-risk HPV oncoproteins are important for their role in the development of HPV-associated cancers and also because they represent simple and tractable research tools that can be used to study tumor suppressor pathways in human cells. The cellular pathways revealed through the studies of oncoproteins encoded by DNA tumor viruses, including the HPVs, have proven to be critical in many non-virus-associated cancers (4). HPV E7 proteins are small proteins of about 100 amino acids (aa). The 40 N-terminal amino acids of E7 are homologous to part of adenovirus E1A conserved region 1 (CR1) and much of CR2 (5), and CR2 includes the conserved LxCxE motif that is responsible for binding to retinoblastoma family proteins. Both CR3 of E1A and the C-terminal half of E7 contain two CxxC motifs that bind zinc ions, but otherwise this part of E7 is not related to E1A.

The widely accepted model of transformation by high-risk HPVs states that E7 proteins bind to retinoblastoma family proteins, including RB1, p107/RBL1, and p130/RBL2, which releases E2F transcription factors and allows passage through the G_1_/S checkpoint (6–8). High-risk E7 proteins additionally promote the degradation of RB1 (9–12). High-risk E6 proteins bind the cellular ubiquitin ligase E6AP to form a complex that targets p53 for proteasome-mediated degradation, thereby blocking signaling through the apoptotic pathways that would otherwise be triggered by RB1 inactivation (13, 14). In addition, high-risk HPV E6 proteins interact with cellular PDZ domain-containing proteins and may target some of them for proteasome-mediated degradation (15). Dysregulated expression of E6 and E7 promotes genomic instability leading to transformation and cancer (16).

There are RB1-independent transforming functions of high-risk E7 proteins that are not explained by this model. High-risk E7 can immortalize primary human foreskin keratinocytes (HFKs) and score in several transformation assays (17–19), but although low-risk HPV E7 proteins also bind RB1, they have no or minimal immortalization or transformation activities (20–22). This difference suggests that retinoblastoma binding is not sufficient for immortalization or transformation. Consistent with this, some mutations in the C terminus of E7 result in proteins that can bind RB1 but not extend the life span of HFKs (23, 24). Also, in other transformation assays, high-risk E7 has RB1-independent activity that maps to the N terminus and/or C terminus of E7 (25–28). A mouse model of cervical cancer confirms that events other than RB1/p107/p130 inactivation must be required for cancer development (29).

E6 and E7 do not have enzymatic activities and thus act through physical interaction with specific host cellular factors. To better understand E6 and E7 activities, we conducted systematic proteomic analyses of interactions between HPV proteins and their host cellular targets using a panel of E6 and E7 proteins from diverse HPV types (30–32). Other laboratories have also conducted similar studies identifying cellular targets of tumor virus proteins, including HPV proteins (33). It is likely that the E6- and E7-interacting proteins identified and validated by these studies have important roles in the HPV life cycle and/or contribute to the transformation activities of E6 and E7. In this article, we focus on the interaction of HPV E7 with the cellular protein PTPN14. Our mass spectrometry studies identified PTPN14 as a binding partner of many of the 17 different HPV E7 proteins included in our study, particularly with the E7 proteins from the genus alpha (mucosal-tropic) viruses (32).

PTPN14 (Pez, PTPD2, or PTP36) is a classical non-transmembrane protein tyrosine phosphatase (PTP) (34). It has an N-terminal FERM (4.1 protein, ezrin, radixin, and moesin) domain that mediates interactions with proteins at the plasma membrane, a C-terminal phosphatase domain, and central PPxY motifs that mediate an interaction with the transcriptional activator YAP1. The gene encoding PTPN14 has been implicated as a cancer gene. An early indication that PTPN14 might have tumor suppressor activity comes from an analysis of somatic mutations in colorectal cancer, in which 87 PTP genes were sequenced and just 6, including the gene coding for PTPN14, were frequently mutated in those tumors (35). Other cancers with PTPN14 mutations include pancreatic adenocarcinomas, basal cell carcinomas, and relapsed neuroblastomas as well as 13.2% of cell lines in the NCI-60 set (36–38). PTPN14 was identified in a screen for PTPs that might function as tumor suppressors in breast cancer development (39).

The association of PTPN14 with several cancers led to our particular interest in PTPN14 as a target of HPV E7 proteins from diverse virus types. In this study, we confirmed the interaction of PTPN14 with several HPV E7 proteins and further discovered that high-risk HPV E7 proteins promote the proteasome-mediated degradation of PTPN14. We map the interaction sites on E7 and on PTPN14 and show that overexpression of the E7-binding fragment of PTPN14 can block the PTPN14-E7 interaction and restore PTPN14 levels. Finally, we find that BPV1 E7 but not HPV1a E7 triggers PTPN14 degradation. Our results are consistent with the model that the targeted degradation of PTPN14 by high-risk E7 could account for some of the retinoblastoma-independent transformation activity of E7 that has been described in the literature for many years.

## RESULTS

### HPV E7 proteins bind PTPN14 and high-risk HPV E7s reduce PTPN14 protein levels.

Our previous proteomic analysis of the cellular interacting proteins of diverse HPV E7 proteins identified the interaction of the cellular protein PTPN14 with 15 different HPV E7 proteins. These PTPN14-binding E7 proteins included all 10 genus alpha E7 proteins and 5 of the 7 genus beta E7 proteins that we examined (32). Twelve of these interactions were considered to be high confidence by the CompPASS algorithm used to assess protein-protein interaction specificity and reproducibility. Independent immunoprecipitation-tandem mass spectrometry (IP-MS/MS) experiments determined that the murine papillomavirus MmuPV1 E7 and the genus gamma HPV197 E7 also bind to PTPN14 (Miranda Grace and Karl Münger, unpublished results).

**FIG 1:**
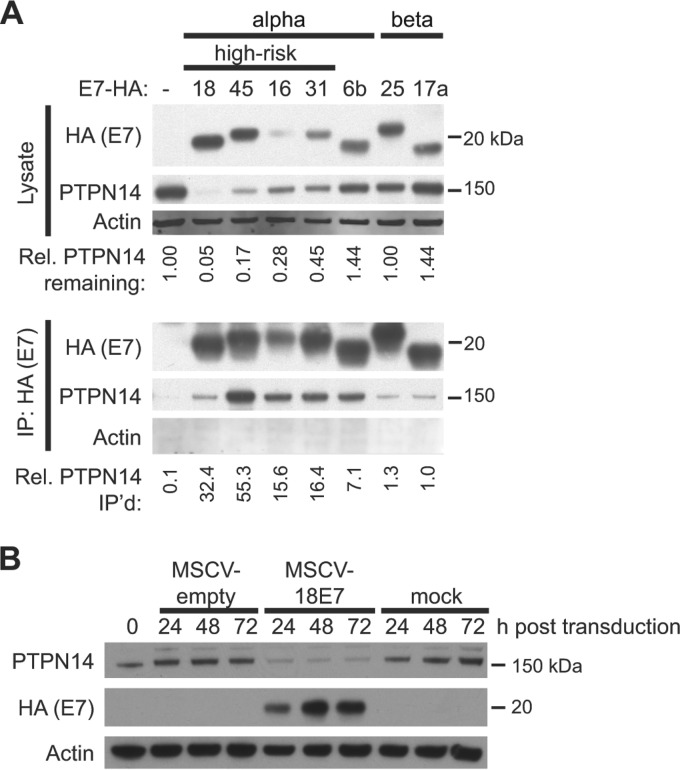
HPV E7 proteins bind to PTPN14, and high-risk HPV E7 proteins reduce PTPN14 levels. (A) Lysates from N/Tert-1 cells expressing E7-Flag-HA were used in immunoprecipitations with HA antibody. Immunoprecipitates were separated by SDS-PAGE and Western blotted using antibodies to HA, PTPN14, and actin. (Top panels) Protein levels in input lysates. Band intensity was quantified with ImageJ, and the numbers indicate the intensity of the PTPN14 band in each lysate lane relative to the actin intensity, normalized to the amount of PTPN14 in the empty vector cells. (Bottom panels) PTPN14 recovered after immunoprecipitation with anti-HA antibody. HA and PTPN14 were detected on film, and actin was detected using the LI-COR imaging system. Band intensity was quantified with ImageJ, and numbers indicate the amount of PTPN14 recovered in the immunoprecipitation relative to the amount of PTPN14 in the lysates, normalized to the amount recovered in the HPV17a E7 IP. (B) N/Tert-1 cells were transduced with a retrovirus encoding HPV18 E7 or with an empty vector control retrovirus or mock transduced and harvested at indicated time points. Steady-state levels of PTPN14, HPV18-Flag-HA, and actin were measured by Western blotting.

To validate this interaction, we performed immunoprecipitation (IP)-Western blot (WB) analysis using a subset of the N/Tert cell lines from our initial E7 study. N/Tert-1 cells are human foreskin keratinocytes immortalized with hTert (40) that we engineered to express epitope-tagged HPV proteins from several different HPV types. Lysates from N/Tert-1 cell lines expressing HPV E7 proteins tagged at the C terminus with a dual Flag and hemagglutinin (HA) epitope tag or control vector-transduced N/Tert cell lines were analyzed by Western blotting for steady-state PTPN14 levels (Fig. 1A, top panels). Unexpectedly, we observed lower PTPN14 protein levels in cell lines expressing E7 proteins from the four high-risk HPV types. Immunoprecipitation of the tagged E7 from these N/Tert cell lysates using anti-HA agarose beads confirmed the binding of endogenous PTPN14 to each of the E7 bait proteins (Fig. 1A, bottom panel). The interaction with PTPN14 was particularly robust for the E7 proteins examined from viruses of the alpha genus of the papillomavirus phylogeny.

To confirm that the reduced PTPN14 levels observed in the N/Tert cells expressing the high-risk E7 proteins was a consequence of E7 expression rather than an artifact that might have resulted from the long-term culture of the N/Tert-1 cell lines used in the proteomics studies, we transduced N/Tert-1 keratinocytes with the retrovirus encoding HPV18 E7 and assessed PTPN14 levels 24 to 72 h posttransduction (Fig. 1B). The reduction in PTPN14 protein was readily detectable 24 h posttransduction, indicating that high-risk HPV E7 is necessary and sufficient for the rapid reduction in PTPN14 levels in N/Tert-1 cells.

### PTPN14 protein levels are low in HPV-positive cervical cancer cells and are restored upon depletion of the HPV E6/E7 early transcript.

**FIG 2:**
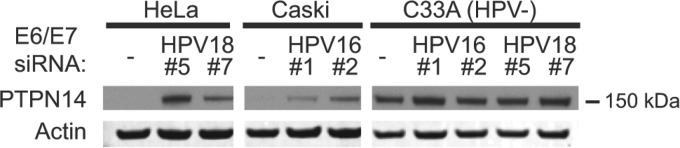
PTPN14 protein levels in cervical cancer cells depend on HPV oncoprotein expression. Cells of the HPV18-positive HeLa, HPV16-positive Caski, and HPV-negative C33A cervical cancer cell lines were transfected with siRNAs targeting the HPV16 E6/E7 and/or HPV18 E6/E7 transcripts at a final concentration of 40 nM. Cells were harvested 72 h posttransfection, and lysates were used for Western blotting with antibodies to PTPN14 and actin.

To determine whether E7-mediated PTPN14 protein reduction also occurs in HPV-positive cervical cancer cell lines, we examined PTPN14 protein in HPV18-positive HeLa cell and HPV16-positive Caski cells (Fig. 2). PTPN14 protein was not detectable in HeLa or Caski cells treated with nontargeting small interfering RNA (siRNA) but was readily detected in HPV-negative C33A cervical cancer cells. Depletion of the bicistronic E6/E7 transcript in either HeLa or Caski cells increased PTPN14 protein levels, but treatment of C33A cells with the same E6/E7 siRNAs did not affect PTPN14 protein levels. Thus, the high-risk HPV oncoproteins were necessary for the reduction of PTPN14 protein levels in cervical cancer cells, and based upon the effect of high-risk HPV E7 expression in the N/Tert-1 cells, we conclude that this resulted from an activity of the E7 oncoprotein.

### The C terminus of HPV16 E7 and the phosphatase domain of PTPN14 are required for the interaction of PTPN14 with E7.

**FIG 3:**
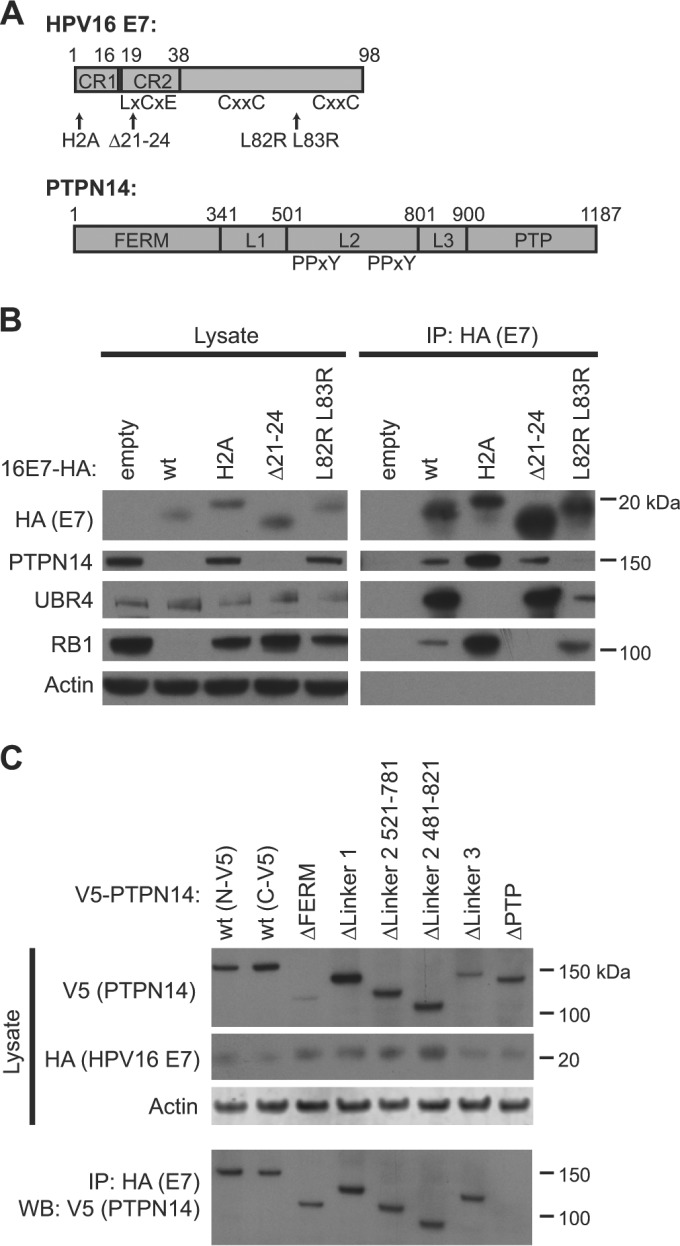
Identification of protein binding sites on HPV16 E7 and on PTPN14. (A) Schematic of HPV16 and PTPN14 domain organization and mutants used in the study (39). (B) N/Tert-1 cells expressing wild-type (wt) HPV16 E7-Flag-HA, one of several mutants of HPV16 (E7), or the empty vector were subjected to immunoprecipitation with HA antibody. Immunoprecipitates were separated by SDS-PAGE and Western blotted using antibodies to HA, PTPN14, UBR4, RB1, and actin. (Left panels) Protein levels in input lysates. (Right panels) Proteins recovered after immunoprecipitation with anti-HA antibody. (C) N/Tert-1 cells expressing wild-type HPV16 E7-Flag-HA were transduced with retroviruses expressing V5-tagged PTPN14 or one of several deletion mutant forms of PTPN14 and were harvested 48 h posttransduction. Cell lysates were subjected to immunoprecipitation with HA antibody. Immunoprecipitates were separated by SDS-PAGE and Western blotted using antibodies to HA, V5, and actin. (Top panels) Protein levels in input lysates. (Bottom panel) V5-tagged PTPN14 recovered after immunoprecipitation with anti-HA antibody.

To determine the regions of E7 and of PTPN14 that contribute to their interaction and to the reduction of PTPN14 protein levels, we performed a series of binding experiments using several well-defined mutants of HPV16 E7 and deletion mutants of PTPN14 (Fig. 3A). We chose to use mutants of HPV16 E7 because of their well-characterized activities in cellular immortalization and transformation assays and their known effects on some protein-protein interactions (8, 25). The Δ21–24 mutation disrupts the LxCxE motif that is required for E7 interaction with RB1 (25). The H2A mutation alters the same conserved amino acid that is altered in the widely used H2P mutant of HPV16 E7 (41) and similarly eliminates the ability of HPV16 E7 to bind to UBR4 (27). The L82R L83R mutant is competent in ras cooperativity assays but somewhat impaired in the ability to transactivate the Ad E2 promoter (25).

We assessed the ability of a panel of HPV16 E7 mutants expressed in stable pools of N/Tert-1 keratinocytes to bind and degrade PTPN14 in IP-WB experiments and selected several of them for further study. Having found that several C-terminal mutations eliminated the ability of HPV16 E7 to bind to PTPN14, we chose to focus on the N- and C-terminal mutations that we predicted would be least disruptive to HPV16 E7, namely, the H2A N-terminal mutant and the L82R L83R C-terminal mutant. Wild-type HPV16 E7 and HPV16 E7 Δ21–24 were also included as controls. This experiment assessed the coimmunoprecipitation of E7 and PTPN14 and the effect of the E7 mutations on the reduction of PTPN14 levels. Both wild-type HPV16 E7 and the RB1-binding mutant of E7 (HPV16 E7 Δ21–24) reduced the steady-state levels of PTPN14 protein, but cells producing HPV16 E7 H2A or HPV16 E7 L82R L83R contained levels of PTPN14 protein equivalent to those in the empty-vector-containing cells (Fig. 3B, left panels). In contrast, only wild-type HPV16 E7 reduced RB1 protein levels. After anti-HA immunoprecipitation, wild-type HPV16 E7 was found to interact with PTPN14, UBR4, and RB1 (Fig. 3A, right panels). HPV16 E7 H2A was deficient in binding to UBR4, HPV16 E7 Δ21–24 was deficient in binding to RB1, and HPV16 E7 L82R L83R was deficient in binding to PTPN14. As previously reported, UBR4 binds to the N terminus of E7 (27, 42), and here we show that PTPN14 binds to the C terminus of E7. We conclude that to reduce PTPN14 levels, E7 must interact with PTPN14 and with the E3 ligase UBR4.

Finally, we used a panel of PTPN14 deletion mutants very similar to a previously described collection of PTPN14 constructs (39). These mutants contain deletions, individually, of the FERM domain (which mediates actin binding and cytoskeletal interaction), the linker 1 region, the linker 2 region (which includes the PPxY motifs required for PTPN14 binding to YAP1), the linker 3 region, and the phosphatase (PTP) domain of PTPN14. N/Tert-16 E7 cells were transduced with retroviruses encoding full-length PTPN14 with an N- or C-terminal V5 tag or PTPN14 deletion mutants with N-terminal V5 tags. HPV16 E7 efficiently coprecipitated each form of PTPN14, except for the PTPN14 ΔPTP protein (Fig. 3B). We conclude that E7 binds to the C-terminal phosphatase domain of PTPN14.

### **Blocking the interaction of PTPN14 with E7 restores PTPN14 levels in E7-producing cells**.

**FIG 4:**
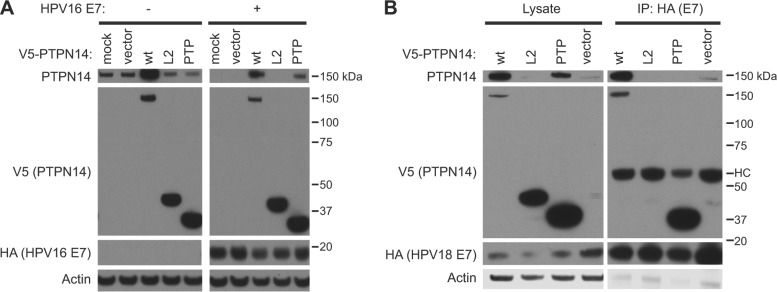
Overexpression of the PTPN14 PTP domain can block the interaction of E7 with full-length PTPN14 and restore PTPN14 levels. (A) N/Tert-1 cells expressing wild-type (wt) HPV16 E7-Flag-HA or the empty vector were mock transduced, transduced with a retrovirus packaged with the empty vector, or transduced with retroviruses expressing wild-type V5-tagged PTPN14, the PTPN14 L2 domain, or the PTPN14 PTP domain and were harvested 48 h posttransduction. Steady-state levels of full-length PTPN14, V5-tagged PTPN14 fragments, HA-tagged E7, and actin were measured by Western blotting. (B) N/Tert-1 cells expressing wild-type HPV18 E7-Flag-HA were transduced with retroviruses expressing wild-type V5-tagged PTPN14, the PTPN14 L2 domain, the PTPN14 PTP domain, or the empty vector and were harvested 48 h posttransduction. Cell lysates were subjected to immunoprecipitation with HA antibody. Immunoprecipitates were separated by SDS-PAGE and Western blotted using antibodies to PTPN14, HA, V5, and actin. (Left panels) Protein levels in input lysates. (Right panels) Proteins recovered after immunoprecipitation with anti-HA antibody. HC, antibody heavy chain.

To further confirm the binding site of E7 on PTPN14 and to determine the effect of interfering with this interaction, we overexpressed either the PTP domain fragment that includes the E7 binding site, full-length PTPN14, or a control linker 2 domain fragment (L2) of PTPN14 that does not bind to E7 (Fig. 4A). In N/Tert-1 cells that do not produce E7, overexpression of full-length wild-type PTPN14 increased the total cellular level of PTPN14 as measured by Western blotting. Neither the L2 nor PTP fragment altered the levels of endogenous PTPN14, as determined by detection with the PTPN14 antibody. In contrast, in cells that produce HPV16 E7, endogenous PTPN14 levels were initially below the limit of detection. Overexpression of either full-length PTPN14 or the E7-binding PTP fragment increased the amount of cellular PTPN14. Restoration of full-length PTPN14 levels by overexpression of an E7-binding fragment of PTPN14 suggested that overexpression of the PTP domain can block the E7-PTPN14 interaction and that this interaction is required for reduction in PTPN14 levels.

To confirm that the PTP domain acts to interfere with the binding of E7 to endogenous PTPN14 and that the increase in PTPN14 levels is observed with other HPV E7s, we transduced N/Tert-HPV18 E7 cells with lentiviruses encoding wild-type PTPN14, the PTPN14 L2 domain, or the PTPN14 PTP domain. Again, full-length PTPN14 was detected in these cells only when it was overexpressed or when the PTP domain was present (Fig. 4B, left panels). A subsequent anti-HA immunoprecipitation showed that although full-length PTPN14 was present in the cells after the introduction of the PTP domain, it was no longer coprecipitated with HPV18 E7 (Fig. 4B, right panels). HPV18 E7 did efficiently coprecipitate the V5-tagged PTP fragment.

### The E7/PTPN14 complex contains UBR4 and is distinct from the E7/RB1 complex.

**FIG 5:**
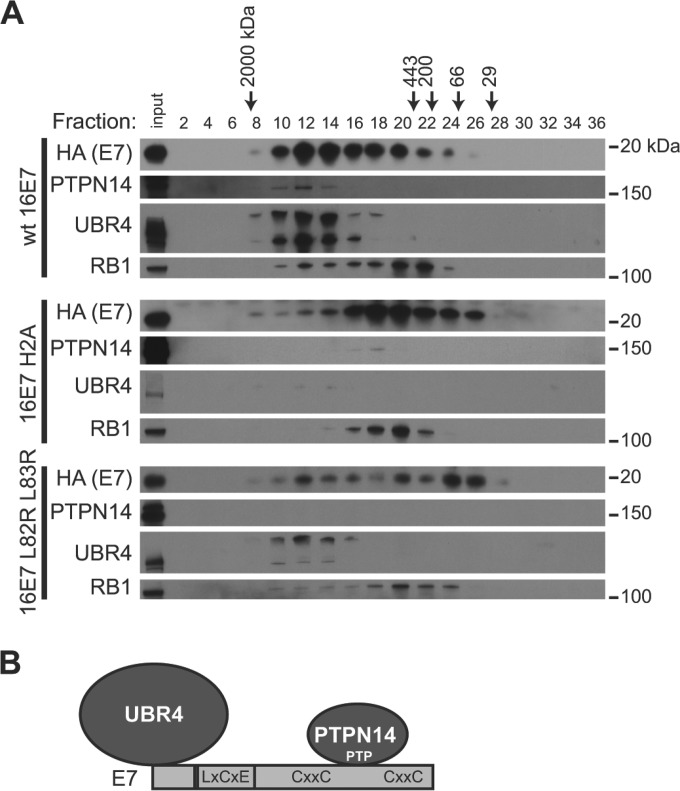
E7 interacts with UBR4 and PTPN14 in a complex distinct from the E7-RB1 complex. (A) Lysates from N/Tert-1 cells expressing wild-type (wt) HPV16 (E7), HPV16 E7 H2A, or HPV16 E7 L82R L83R were fractionated on a Superose 6 column using a GE Akta Pure high-performance liquid chromatography (HPLC) column. Five hundred-microliter fractions were collected, and alternate fractions were used for anti-HA immunoprecipitation. Immunoprecipitates were separated by SDS-PAGE and Western blotted using antibodies to PTPN14, HA, UBR4, and RB1. Unfractionated lysate (input) is included on each gel as a control. (B) Schematic of E7/PTPN14/UBR4 complex and approximate interaction sites.

To characterize the E7/PTPN14 complex, lysates from N/Tert-1 cells expressing wild-type HPV16 E7 or the PTPN14 degradation-deficient mutants HPV16 E7 H2A and HPV16 E7 L82R L83R were separated into 500-µl fractions by gel filtration chromatography on a Superose 6 column. Even-numbered fractions were used as the starting material in anti-HA immunoprecipitations to recover E7 and its associated proteins, and immunoprecipitates from each fraction were analyzed by Western blotting. In cells producing wild-type HPV16 E7, we found at least two separate protein complexes containing E7. One is a high-molecular-mass complex that eluted with a peak around fraction 12 and contained PTPN14, UBR4, and a small amount of RB1, and a second complex eluted with a peak around fractions 20 to 22 and contained E7 and RB1 (Fig. 5A, top panels). We hypothesize that although E7 bound to PTPN14 and UBR4 may still engage RB1, the majority of E7-RB1 binding occurs in a separate, smaller complex and with a pool of E7 that is not also bound to PTPN14 or UBR4 (Fig. 5B).

Next we fractionated lysates from PTPN14 degradation-deficient mutant cell lines and found that mutant E7 proteins bind to PTPN14 and/or RB1, consistent with separate E7/PTPN14/UBR4 and E7/RB1 complexes. The HPV16 E7 H2A mutant does not interact with UBR4 (Fig. 3B). Consequently, in the gel filtration experiment, the bulk of E7 was shifted into lower-molecular-mass fractions that do not contain UBR4 (Fig. 5A, middle panels). Faint binding to PTPN14 was still observed in fraction 18, corresponding to the peak of E7 elution. The E7-Rb complex was close to the same size as the mutant E7/PTPN14 complex, although its peak is in fraction 20, and we speculate that the two complexes remain distinct from one another. In contrast, the HPV16 L82R L83R mutant retained the ability to engage UBR4, but did not bind PTPN14 (Fig. 3B). When this mutant was used in the gel filtration assay, E7 L82R L83R eluted more slowly from the column than its wild-type counterpart, but the mutant E7/UBR4 complex was clearly distinct from the E7/RB1 complex (Fig. 5A, bottom panels).

### High-risk HPV E7 recruits UBR4 to direct the proteasome-mediated degradation of PTPN14.

**FIG 6:**
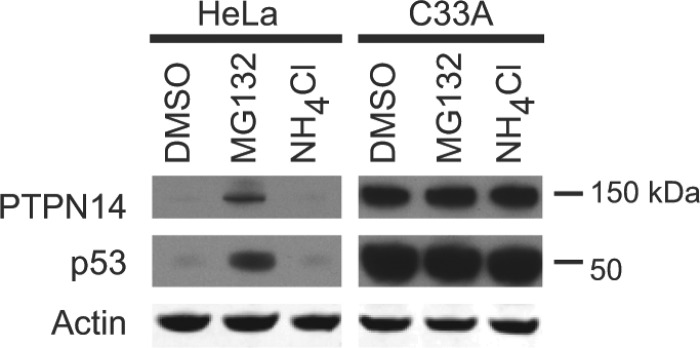
PTPN14 is degraded by the proteasome in HPV-positive cervical cancer cells. HPV18-positive HeLa cells and HPV-negative C33A cervical cancer cells were treated for 8 h with 30 µM MG132, 10 mM NH_4_Cl, or DMSO as a control. Lysates were separated by SDS-PAGE and Western blotted using antibodies to PTPN14, p53, and actin.

To determine the pathway by which PTPN14 is degraded in the presence of E7, we examined whether treatment with MG132, a proteasome inhibitor, or NH_4_Cl, which inhibits lysosome acidification, had any impact on PTPN14 protein levels (Fig. 6). In HeLa cells, which express HPV18 E7, PTPN14 was below the limit of detection. PTPN14 levels increased upon treatment with MG132 but not with NH_4_Cl treatment. It is well established that p53 is ubiquitylated by the E3 ligase E6AP and targeted for degradation by the proteasome in HPV-positive cancer cells (43), and the effects of the drug treatments on p53 were similar to their effects on PTPN14. In HPV-negative C33A cervical cancer cells, neither PTPN14 nor p53 levels were altered by MG132 or NH_4_Cl treatment.

**FIG 7:**
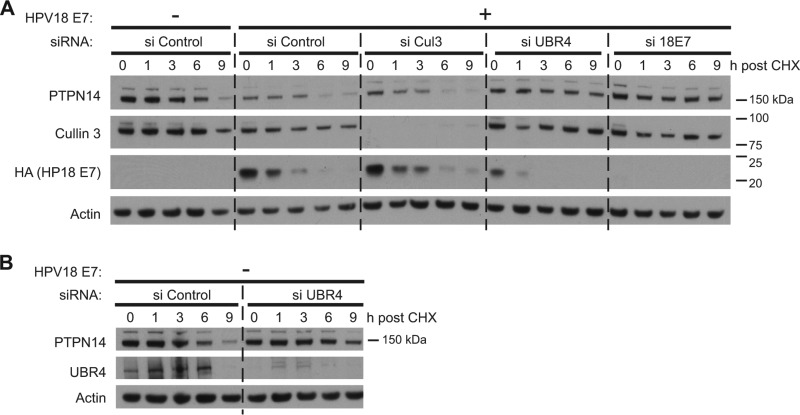
HPV18 E7-mediated PTPN14 degradation requires the E3 ubiquitin ligase UBR4. (A) N/Tert cells were transfected with siRNAs targeting cullin 3, UBR4, or HPV18 E7 or with a nontargeting control (si Control). Twenty-four hours posttransfection, cells were transduced with a retrovirus expressing HPV18 E7 or with the empty vector control retrovirus. Forty-eight hours post-retrovirus transduction, cells were treated with 40 µg/ml cycloheximide (CHX) and harvested at indicated time points post-cycloheximide treatment. Equal amounts of protein lysates were separated by SDS-PAGE and Western blotted using antibodies to PTPN14, cullin 3, HA, and actin. (B) N/Tert cells were transfected with siRNA targeting UBR4 or with a nontargeting control (si Control). Twenty-four hours posttransfection, cells were transduced with an empty vector control retrovirus. Forty-eight hours post-retrovirus transduction, cells were treated with 40 µg/ml cycloheximide and harvested at indicated time points post-cycloheximide treatment. Lysates were separated by SDS-PAGE and Western blotted using antibodies to PTPN14, UBR4, and actin. The same lysates were run in the first five lanes of the gels in panels A and B. The experiment was repeated four times, and a representative experiment is shown.

Based on the results of the mapping experiments shown in Fig. 3 and the gel filtration data shown in Fig. 5, we hypothesized that in the presence of high-risk HPV E7, PTPN14 is targeted for degradation by the proteasome and that high-risk E7 must engage both UBR4 and PTPN14 in order to reduce PTPN14 levels. UBR4 is an E3 ubiquitin ligase that binds E7 and has been shown to have some role in E7-mediated transformation (27, 42, 44) that has not been completely elucidated. Since E3 ligase activity is a critical component of the pathway that targets proteins for proteasome-mediated degradation, we hypothesized that UBR4 might be required for the proteasome-mediated degradation of PTPN14 in the presence of E7. To test this hypothesis, N/Tert-1 cells were transfected with control siRNA or one of several other siRNAs and then transduced with control or HPV18 E7 retrovirus. At 72 h post-siRNA transfection, the cells were treated with cycloheximide to block *de novo* protein synthesis and were harvested in a time course experiment. We found that in the absence of HPV18 E7, PTPN14 protein levels declined after cycloheximide treatment (Fig. 7A; see Fig. S1 in the supplemental material), with a half-life of about 5.5 h. In the presence of HPV18 E7, the amount of PTPN14 protein present at the beginning of the experiment was lower than in the absence of HPV18 E7 and was degraded rapidly with little residual protein at the end of the time course.

To determine whether UBR4 or cullin 3 (CUL3) might be required for this reduction in PTPN14 protein levels, we assessed PTPN14 stability in cells with siRNA-mediated depletion of UBR4 or of CUL3. CUL3 is the scaffolding component of many E3 ligases, and it interacts with several HPV E7 proteins (32), but CUL3 depletion did not significantly change the amount of PTPN14 or increase its half-life in this assay. In contrast, siRNA depletion of UBR4 increased the amount of PTPN14 present in the HPV18 E7-expressing cells at the 0-h time point and extended the half-life of PTPN14 to almost 10 h in the cycloheximide chase. Depletion of HPV18 E7 itself also restored PTPN14 levels and extended its half-life.

To confirm that UBR4 targets PTPN14 for degradation only in the presence of E7, we examined UBR4’s effect on PTPN14 stability in a cycloheximide chase experiment in cells without E7 (Fig. 7B). When E7 was not present, UBR4 siRNA treatment had little effect on the starting level of PTPN14 or on its stability. We conclude that in the presence of HPV18 E7, UBR4 is required for the degradation of PTPN14. A cullin 2-LRR1 ubiquitin ligase has been previously shown to target PTPN14 for proteasome-mediated degradation in the absence of E7 (39).

### Human and animal papillomavirus E7 proteins vary in their ability to bind UBR4 and target PTPN14 for degradation.

RB1 binding and degradation have long been recognized to contribute to the cellular transformation activity of the high-risk HPV E7 proteins. However, it is equally well appreciated that high-affinity RB1 binding by E7 does not correlate with the ability of E7 proteins to act in cellular transformation assays. For example, HPV1a E7 binds RB1 with high affinity but does not transform (45, 46). In contrast, BPV1 E7 does not bind RB1 but has activity in transformation assays when coexpressed with E5 and E6 (47). Wild-type BPV1 E7 is also required for transformation in the context of the complete BPV1 genome (48, 49).

**FIG 8:**
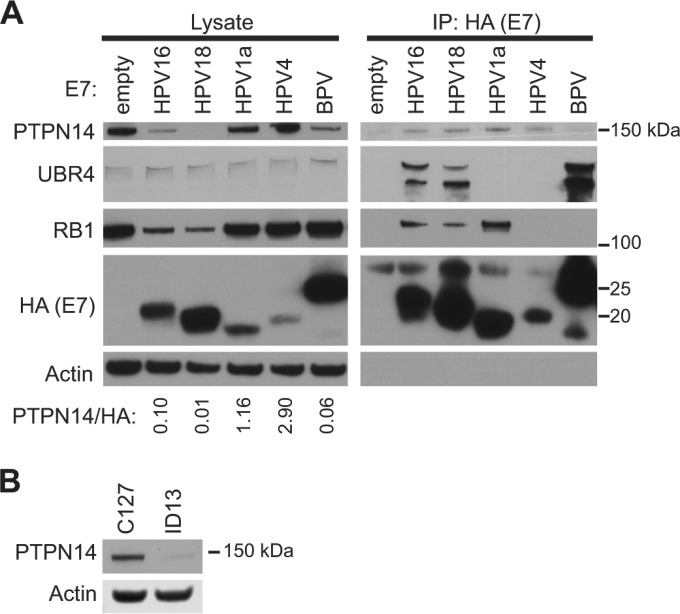
Papillomavirus E7 proteins vary in their ability to bind PTPN14 and target PTPN14 for degradation. (A) N/Tert-1 cells expressing one of several HPV E7 proteins or BPV1 E7 protein tagged with Flag and HA were subjected to immunoprecipitation with HA antibody. Immunoprecipitates were separated by SDS-PAGE and Western blotted using antibodies to HA, PTPN14, UBR4, RB1, and actin. (Left panels) Protein levels in input lysates. (Right panels) Proteins recovered after immunoprecipitation with anti-HA antibody. PTPN14 and HA protein amounts were quantified using ImageJ. The ratio of PTPN14 protein amount relative to E7-HA protein amount in the E7-expressing cell lines is indicated. (B) PTPN14 and actin protein levels in C127 and ID13 cell lysates were measured by Western blotting.

To determine whether PTPN14 degradation might better correlate with E7 transformation activity than does RB1 binding, we assessed the ability of several HPV E7 proteins from diverse genera as well as BPV1 E7 to target PTPN14 for degradation. In IP-WB experiments using E7 proteins stably expressed in N/Tert-1 keratinocytes, we observed that BPV1 E7, but not HPV1a E7 nor the genus gamma HPV4 E7, was able to reduce the steady-state level of PTPN14 (Fig. 8A). Consistent with this result, we observed that neither HPV1a nor HPV4 E7 coimmunoprecipitated UBR4. We hypothesize that these E7 proteins are unable to target PTPN14 for degradation because they are unable to robustly engage UBR4. BPV1 E7 efficiently bound UBR4 and consequently was able to reduce PTPN14 levels. Thus, PTPN14 degradation correlates better with the ability of E7 to act in transformation assays than does RB1 degradation. We note that further experiments will be required to determine the relative contributions of PTPN14 degradation versus retinoblastoma inactivation in these assays.

PTPN14 was difficult to detect on a Western blot after immunoprecipitation of BPV1 E7 likely because of its efficient degradation in the presence of this E7. Consistent with the ability of BPV1 to reduce PTPN14 levels in N/Tert-1 cells, we observed that PTPN14 was readily detectable by Western blotting in mouse C127 cells but not in ID13 cells (Fig. 8B). ID13 cells are C127 cells transformed with the BPV1 genome, and they produce BPV1 E7.

## DISCUSSION

Decades of study have helped to explain how high-risk E6 and E7 act in immortalization and transformation assays, yet there are still open questions regarding the mechanisms by which these proteins dysregulate host cellular functions. For more than 25 years, there has been evidence in the literature that high-risk HPV E7 proteins have retinoblastoma-independent immortalization and transformation activities (24, 46). Many studies support the idea that N- and/or C-terminal regions of E7 are important for its retinoblastoma-independent contributions to transformation, and at least one report suggested that this activity could require interaction with a yet unidentified cellular protein (23). Other studies hinted at what cellular factor might be involved. For example, the E3 ubiquitin ligase UBR4 binds to the N terminus of E7 and is required for the anchorage-independent growth of HPV-positive cervical cancer cell lines and for the ability of BPV1 E7 to inhibit anoikis (27, 42, 44), but in the absence of a target for ubiquitylation by UBR4, the mechanistic basis of these observations was unclear.

Our previous report on the E7 protein interaction data focused on aspects of E7 function related to RB degradation. We described the cullin 2/Zer1 E3 ubiquitin ligase complex that is required for the HPV16 E7-mediated degradation of RB1, and this together with an earlier study established the mechanism of RB1 degradation by HPV16 E7 (32, 50). Next we chose to focus on proteins that could help to explain the retinoblastoma-independent immortalization and transformation activity of high-risk HPV E7 and protein interactions that would distinguish high-risk E7 from low-risk E7.

PTPN14 degradation by E7 is an excellent candidate to account for the data supporting the retinoblastoma-independent transformation activity of E7. High-risk E7 is necessary and sufficient for PTPN14 degradation, and PTPN14 degradation is not an activity of low-risk E7 (Fig. 1 and 2). PTPN14 degradation requires the N- and C-terminal regions of E7 that are known to be important in retinoblastoma-independent transformation (Fig. 3). Our results fit with the retinoblastoma-independent nature of the missing transformation function, since RB1 binding is not necessary for PTPN14 degradation (Fig. 3), and E7 interacts with PTPN14 and UBR4 in a complex that is largely distinct from the RB1-containing complex (Fig. 5). In addition, our results are consistent with the previously identified role of UBR4 in retinoblastoma-independent transformation, since the degradation of PTPN14 in the presence of E7 requires UBR4 (Fig. 7). Our mapping studies suggest that this is because the N terminus of E7 binds UBR4 and the C terminus binds PTPN14 (Fig. 3). Although low-risk and non-cancer-associated E7 proteins engage both PTPN14 and UBR4, we hypothesize that the more robust interaction of high-risk E7 with both proteins (Fig. 1) (32) is responsible for the high-risk E7-specific degradation of PTPN14. We have not determined whether the interaction of E7 with UBR4 increases its affinity for PTPN14, or vice versa.

We also see a close correlation between PTPN14 degradation and RB-independent transformation in two assays. In a previously published study, we found that HPV16 E7 and some mutants of HPV16 E7 have activity in an assay of retinoblastoma-independent transformation (51). The HEK TER cells used in that assay produce simian virus 40 (SV40) large T antigen, which efficiently inactivates RB1, yet E7 retains some ability to transform. That retinoblastoma-independent transformation activity mapped to the N and C termini of HPV16 E7, which we now know are required for PTPN14 degradation. The second correlation is in this article, in which we examined PTPN14 degradation by two E7 proteins for which there has historically not been a good connection between RB1 binding and transformation. One is HPV1a E7, which binds RB1 with high affinity but does not transform (45, 46), and the other is BPV1 E7, which does not bind retinoblastoma proteins but cooperates with BPV1 E5 and E6 in transformation assays (47–49). We found that the previously established ability of these proteins to transform is correlated with their ability to reduce PTPN14 levels (Fig. 8). PTPN14 was degraded in the presence of HPV16 E7, HPV18 E7, and BPV1 E7, each of which has activity in transformation assays. PTPN14 was not degraded in the presence of HPV1a E7, which does not contribute to transformation even though it efficiently binds RB1. Other published data are also consistent with a link between PTPN14 degradation and retinoblastoma-independent transformation. HPV18 E7 DNA was 3 to 4 times more efficient than HPV16 DNA at altering keratinocyte differentiation (52), and HPV18 E7 is the most efficient at targeting PTPN14 for degradation in our assays (Fig. 1). We plan further experiments to determine the importance of PTPN14 degradation relative to retinoblastoma inactivation in transformation assays, but for the high-risk HPV E7 proteins, we anticipate that both activities contribute to transformation by E7.

It is interesting to consider how PTPN14 degradation might address the limitations of the current HPV-associated transformation model. A revised model of transformation has proposed that an initial oncogenic event occurs when high-risk E7 proteins are expressed in cells (53). This leads to the induction of p16^INK4A^, a negative regulator of the cell cycle that is upregulated in high-risk HPV-associated cancers. The induction of p16^INK4A^ and subsequently p53 would normally lead to cell cycle arrest and oncogene-induced senescence mediated by RB1. To avoid this, E7 proteins inactivate RB1 and bypass the senescence that would otherwise result from the initial oncogenic hit. This model could account for the RB1-independent transformation functions of high-risk HPVs and could explain why high-risk but not low-risk E7 proteins are oncogenic, but the initial oncogenic event has not been identified. Our future studies will examine the possibility that PTPN14 degradation provides this initial oncogenic event.

Finally, there are other binding partners of E7, and we do not discount the possibility that they may also act in RB-independent transformation by E7. p190RhoGAP binds to the C terminus of several HPV E7 proteins, although this interaction is not specific for high-risk HPVs (54). We also note that there could be more than one mechanism by which HPV E7 proteins act to reduce PTPN14 levels. PTPN14 is a target of miR-21, and this microRNA (miRNA) downregulates PTPN14 and PTEN at the mRNA and protein levels (55). E7 is responsible for the upregulation of miR-21 in cervical cancer (56–59), so the E7-induced increase in miR-21 could lead to a decrease in PTPN14 expression in E7-positive cells.

PTPN14 is an appealing candidate to contribute to transformation by E7 because of its involvement in cell growth controls. PTPN14 can act as a negative regulator of YAP1 in the Hippo signaling pathway, which regulates proliferation and apoptosis (39, 60, 61). YAP1 and its homolog TAZ activate TEAD-dependent promoters that control proproliferative and antiapoptotic genes. The published connection between Hippo signaling, which involves PTPN14, and anoikis (62) could also help to explain why UBR4 was previously found to be required for the E7-mediated inhibition of anoikis (44). YAP affects other signaling pathways, including the Wnt, transforming growth factor β (TGF-β), and Notch pathways (63), and so there is potential for PTPN14 to impact them as well, but this has not been studied. Negative regulation of YAP1 could explain the tumor suppressor functions of PTPN14, but in contrast, a recent publication showed that PTPN14 promotes aberrant cell growth in a breast cancer model (64). As for many PTPs, cell type and signaling context may significantly dictate the downstream effects of PTPN14 activity. Notably, PTPN14’s role in YAP repression appears not to require its phosphatase activity, and the PTPN14-YAP interaction appears to be the major source of PTPN14 tumor-suppressive activity (39). Future studies will test the effect of E7-mediated PTPN14 degradation on YAP1 and on the many pathways with which it is involved.

Our results demonstrate that high-risk HPV E7 bind and target PTPN14 for proteasome-mediated degradation. This degradation requires UBR4 and can be inhibited by blocking the interaction between E7 and PTPN14. The ability of E7 to target PTPN14 for degradation maps to the N and C termini of E7 and is independent of RB1 binding by E7, making it consistent with previous suggestions in the literature that there is a retinoblastoma-independent transformation activity of E7. PTPN14 is mutated in several cancers, and our subsequent studies will investigate the effect of restoration of PTPN14 levels in HPV-positive cancer cells. We also plan to investigate the effect of the E7-PTPN14 interaction on host cellular pathways, including Hippo signaling and other pathways impacted by YAP.

## MATERIALS AND METHODS

### Cloning and plasmids.

The murine stem cell virus (MSCV)-empty vector and MSCV-HPV18E7-Flag-HA plasmids used in transient transduction experiments have been previously described (32, 65). MSCV vectors encoding HPV1a E7, HPV4 E7, BPV1 E7, and deletion and substitution mutant forms of HPV16 E7 were constructed as previously described (32). The PTPN14 deletion mutant and PTPN14 subfragment overexpression constructs were based on a previously published collection of PTPN14 deletion mutants (39), which were a gift from Junjie Chen (University of Texas M. D. Anderson Cancer Center) and served as the starting material for subcloning into Gateway cloning-compatible MSCV retroviral and/or phage lentiviral vectors. Howley lab plasmid database numbers for each of the plasmids used in the study are listed in Table S1 in the supplemental material. The PTPN14 deletion mutant constructs are missing the following amino acids: ΔFERM, aa 1 to 341; ΔLinker 1, aa 341 to 501; ΔLinker 2 version 1, aa 521 to 781, ΔLinker 2 version 2, aa 481 to 821; ΔLinker 3, aa 801 to 900; and ΔPTP, aa 900 to 1187. The overexpressed L2 fragment corresponds to PTPN14 aa 501 to 801, and the overexpressed PTP fragment corresponds to PTPN14 aa 900 to 1187.

### Cell lines and transductions.

N/Tert-E7 cell lines and their propagation have been previously described, and new N/Tert-1 cell lines were established and selected in the same way (32). HeLa, Caski, C33A, C127, and ID13 cells were grown in Dulbecco’s modified Eagle’s medium (DMEM) with 10% fetal bovine serum and 100 U/ml penicillin and 100 µg/ml streptomycin. For MG132 and NH_4_Cl treatments, cells were treated with 30 µM MG132, 10 mM NH_4_Cl, or dimethyl sulfoxide (DMSO) control in culture medium for 8 h prior to harvest. For cycloheximide treatment, cells were treated with 40 µg/ml cycloheximide in culture medium and harvested at the indicated time points.

### siRNA transfections.

N/Tert-1, HeLa, Caski, and C33A cells were transfected with siRNAs as previously described (32, 66) using Dharmafect 2 transfection reagent for N/Tert-1 and C33A cells and Dharmafect 1 transfection reagent for HeLa and Caski cells. The siRNAs used in the study were purchased from Dharmacon and are nontargeting siRNA 1 (D-001810-01), CUL3 (J-010224-09), UBR4 (J-014021-09), two custom-designed siRNAs targeting the HPV16 E6/E7 transcript, and two custom-designed siRNAs targeting the HPV18 E6/E7 transcript. The sequences of the custom siRNAs are as follows: HPV16 1, CAACAUUAGAACAGCAAUAUU; HPV16 2, GGACAGAGCCCAUUACAAUUU; HPV18 5, GGAAGAAAACGAUGAAAUAUU; and HPV18 7, GCUAGUAGUAGAAAGCUCAUU. SiGLO red (D-0011630-02) was used to visualize efficient transfection in a control well.

### Western blots, antibodies, and immunoprecipitations.

Western blots were performed as previously described (32) using NuPAGE (Invitrogen) gels and transfer to polyvinylidene difluoride (PVDF). Membranes were blocked in 5% nonfat dried milk in TBST (Tris-buffered saline [pH 7.4] with 0.05% Tween 20) and then incubated with primary antibodies as follows: RB1 (Calbiochem/EMD), UBR4 (gift of Yoshihiro Nakatani, Dana-Farber Cancer Institute [67]), CUL3 (Bethyl), actin (Millipore), V5 (Invitrogen), p53 (Santa Cruz Biotechnology), or PTPN14 (Sigma-Aldrich, R&D Systems, or Cell Signaling Technology). Membranes were washed in TBST and incubated with horseradish peroxidase (HRP)-coupled anti-mouse or anti-rabbit antibodies or an Alexa 680-coupled anti-mouse antibody and detected using Western Lightning chemiluminescent substrate or a LI-COR infrared imaging system. HA-tagged proteins were detected using an HA antibody conjugated to HRP (Roche) and visualized on film. For anti-HA immunoprecipitations, HA-tagged proteins were immunoprecipitated and processed for Western blotting as previously described (32).

### Gel filtration chromatography.

Gel filtration chromatography was performed essentially as previously described (68). Briefly, frozen pellets of N/Tert-E7 cells were resuspended in mammalian cell lysis buffer (MCLB: 50 mM Tris [pH 7.8], 150 mM NaCl, 0.5% NP-40) in the presence of protease and phosphatase inhibitors (Roche Complete, EDTA-free protease inhibitor cocktail and 25 mM sodium fluoride, 1 mM sodium orthovanadate, 5 mM β-glycerophosphate). The lysate was incubated on ice for 15 min then clarified by centrifugation in a refrigerated microcentrifuge for 10 min at top speed. The supernatant was further clarified using 0.45-µm Durapore PVDF spin filters (Millipore). Approximately 7 mg of total cellular protein was applied to a Superose 6 10/300-Gl column run on an Akta Pure fast protein liquid chromatography (FPLC) column (GE Healthcare) with MCLB as the running buffer. The injection volume was 500 µl, the flow rate was 0.5 ml/min, and 0.5-ml fractions were collected from 0.2 column volume to 1.5 column volumes. The molecular masses corresponding to individual fractions were estimated by loading 1 mg of individual protein standards from a Sigma-Aldrich gel filtration marker kit for protein molecular masses from 29,000 to 700,000 Da on the column in the same way and recording the fraction in which the peak eluted. Even-numbered fractions were used as the starting material in anti-HA immunoprecipitations.

## SUPPLEMENTAL MATERIAL

Figure S1  Quantification of PTPN14 levels in cycloheximide chase experiments. ImageJ software was used to quantify the intensity of the bands in the PTPN14 Western blots shown in Fig. 7. The graphs indicate the amount of PTPN14 protein present at each point post-cycloheximide addition, relative to the amount of PTPN14 present in the no-E7/nontargeting siRNA control (si Control) at the 0-h time point. The PTPN14 half-life for each condition was calculated based on a linear regression of each data set shown here. Download Figure S1, EPS file, 0.4 MB

Table S1  Plasmids used in this study. Table S1, XLS file, 0.02 MB
